# Synthesis of a Novel Rigid Semi-Alicyclic Dianhydride and Its Copolymerized Transparent Polyimide Films’ Properties

**DOI:** 10.3390/polym14194132

**Published:** 2022-10-02

**Authors:** Yao Wang, Xiangfu Liu, Jiulin Shen, Jianqiao Zhao, Guoli Tu

**Affiliations:** Wuhan National Laboratory for Optoelectronics, Huazhong University of Science and Technology, 1037 Luoyu Road, Wuhan 430074, China

**Keywords:** colorless polyimide, new dianhydride, coefficient of thermal expansion, glass transition temperatures

## Abstract

A new series of colorless polyimides (CPIs) with outstanding thermal properties and mechanical properties were fabricated by the copolymerization of a novel dianhydride and 4,4′-(hexafluoroisopropylidene)diphthalic anhydride (6FDA) with 2,2′-bistrifluoromethyl benzidine (TFDB). The novel dianhydride, 10-oxo-9-phenyl-9-(trifluoromethyl)-9,10-dihydroanthracene-2,3,6,7-tetraacid dianhydride (3FPODA), possessed a rigid semi-alicyclic structure, –CF_3_ and phenyl side groups, and an active carbonyl group. Benefitting from the special structure of 3FPODA, the glass transition temperatures (T_g_) of the new CPIs improved from 330 °C to 377 °C, the coefficient of thermal expansion (CTE) decreased from 46 ppm/K to 24 ppm/K, and the tensile strength (T_S_), tensile modulus (T_M_), and elongation at break (E_B_) increased from 84 MPa to 136 MPa, 3.2 GPa to 4.4 GPa, and 2.94% to 4.13% with the increasing amount of 3FPODA, respectively. Moreover, the active carbonyl group of the 3FPODA could enhance the CPI’s adhesive properties. These results render the new dianhydride 3FPODA an ideal candidate monomer for the fabrication of high-performance CPIs.

## 1. Introduction

The Polyimides (PIs), a group of high performance polymers with excellent thermal and mechanical properties, as well as a low dielectric constant and outstanding chemical resistance [1,2,3,4,5,6], have been extensively used as flexible substrates in flexible copper clad laminates (FCCL), flexible printed circuit boards (FPCBs), and optoelectronics devices [7,8,9,10,11,12]. FPCBs are generally composed of copper (Cu) foil laminated with PI film, which can enhance the mechanical strength and tenacity of FPCBs so that the electric conductivity properties are retained during bending, sliding, friction and physical shock, etc [13]. To meet the process requirements, a PI is expected to possess extraordinary thermal stability (T_g_ > 350 °C), a low coefficient of thermal expansion (CTE) to match that of copper (17 ppm/K), excellent mechanical properties, and good adhesive properties. However, with the development of the display industry, FPCBs have led to further requirements for PI with respect to optical properties.

The conventional aromatic PIs display a yellow or brown color caused by the formation of the charge transfer complex (CTC) effect between the donor diamine and the acceptor dianhydride moieties [14,15,16]. In order to obtain colorless polyimide (CPI) films, different endeavors have been proposed to achieve the modification of traditional aromatic PI, such as the incorporation of flexible groups [17,18,19], large-scale substituents [20,21,22,23], ring structures [24,25,26], fluorine-containing structures [27,28,29], and twisted or non-planar structures [30,31] in its backbone to limit the formation of the CTC effect to improve the film’s transparency. Generally, the decreased CTC effect of PI films will degrade other properties, including its thermal stability and mechanical properties [32]. Therefore, balancing the optical properties with thermal stability and mechanical properties to fabricate CPI with outstanding comprehensive properties is still a challenge.

Up to now, many new diamine monomers have been developed to prepare CPI. To our knowledge, TFDB is the most used diamine for the fabrication of CPI, which has been commercialized. However, there are relatively few studies on dianhydride monomers, which is the other key monomer for CPI. 6FDA, possessing two –CF_3_ groups, is a well-known dianhydride monomer for CPI. The CPIs based on 6FDA have been reported to present excellent optical properties (T_500_ about 85%) due to the restriction of the formation of the CTC effect caused by the hexafluoroisopropyl group. In addition, the fluorinated PIs could enable other advantages such as good thermo-oxidative stability, a low refractive index, and birefringence. However, the CPI films based on 6FDA display a high CTE (49 ppm/K), an insufficiently high T_g_ (<350 °C), and poor mechanical properties owing to the weak rigidity of their molecular chain [33]. 1,2,4,5-cyclohexanetetracarboxylic dianhydride (HPMDA), as an aliphatic dianhydride monomer, can significantly reduce the CTC effect and improve the film transmittance due to its six-membered ring structure. Moreover, this structure retains a certain rigidity; as a result, the HPMDA-based CPIs display a T_g_ over 350 °C [34]. However, the CTE of these CPIs are higher than 50 ppm/K and cannot satisfy the requirements of FPCBs. In 2017, we reported a new symmetric structure of dianhydride 9,10-difluoro-9,10-bis(trifluoromethyl)-9,10-dihydroanthracene-2,3,6,7-tetracarboxylic acid dianhydride (8FDA) [35]. The CPI film based on 8FDA has outstanding optical and thermal properties (T_g_ = 401 °C, CTE = 14.49 ppm/K). However, the mechanical properties of the CPI with 8FDA are not strong enough with a quite low tensile modulus (T_M_) of 1.2 GPa, which is much less than the 3.0 GPa of the common PI films. Moreover, these three types of CPIs possess poor interfacial activity, without any active group to react with the groups of binders. Therefore, the problem of preparing appropriate CPIs for transparent FPCBs still persists.

In this study, a new rigid, semi-alicyclic dianhydride 3FPODA, containing –CF_3_, phenyl side groups, and a carbonyl group, were designed and synthesized combining the advantages of the above dianhydrides for CPIs. On the one hand, the introduction of a rigid semi-alicyclic structure and a carbonyl group on the 6FDA are expected to enhance the rigidity and linearity of the CPIs to improve their thermal and mechanical properties [35]. Furthermore, the incorporation of the carbonyl group could improve the adhesive properties and interfacial activity of the CPIs because of its reactivity and strong transfer charge action. On the other hand, the –CF_3_ and phenyl side group could further enhance the thermal properties [36,37,38]. Moreover, the –CF_3_ and phenyl side groups can increase the distance between molecular chains to reduce the chain packing to hinder the formation of the CTC effect [39]. These features could remarkably improve the optical and solubility properties of CPI films. However, the carbonyl group will lead to the formation of a conjugation with the two benzene rings. Therefore, we copolymerized different proportions of 3FPODA and 6FDA to balance the optical properties and thermal properties of CPIs, and investigated their optical, thermal, mechanical, and surficial properties in detail.

## 2. Materials and Methods

### 2.1. Materials

6FDA was obtained from Aladdin (Shanghai, China). TFDB and dry N, N-dimethylacetamide (DMAc) were obtained from Wuhan Imide (Wuhan, China). Dry tetrahydrofuran (THF) and trifluoromethanesulfonic acid (TFSA) were purchased from Energy Chemical (Shanghai, China). Other solvents were all obtained from Sinopharm Chemical Reagent Co., Ltd. (Shanghai, China).

### 2.2. Instrumentation

^1^H, ^13^C, and ^19^F NMR spectra were all created via Bruker DRX-600 spectrometer (Karlsruhe, Germany) with chloroform-*d* (CDCl_3_), dichloromethane-*d*_2_ (CD_2_Cl_2_), and dimethylsulfoxide (DMSO-*d*_6_) as deuterated solvents. IR spectra were all measured by PerkinElmer SP one FT-IR (Waltham, MA, USA). Wide-angle X-ray diffraction (WAXD) measurement were carried out on Bruker Bede XRD Di (Karlsruhe, Germany). The optical properties of PI films were recorded on Shimadzu UV-2450 (Kyoto, Japan) from 300 to 800 nm. The refractive index of PI films was measured by ME-Lellipsometer (Wuhan, China). Thermogravimetric analysis (TGA) measurements were performed on PerkinElmer TGA-2 (Waltham, MA, USA) under nitrogen conditions at rate of 10 °C min^−1^ from room temperature to 800 °C. Dynamic mechanical analyses (DMA) were carried out on PerkinElmer DMA Q800 using tensile mode at a frequency of 1 Hz. Coefficient of thermal expansion (CTE) values were measured by thermal mechanical analysis (TMA) Q400 (USA). Mechanical properties were all measured by stretching machine. Contact angles were measured at room temperature with deionized water as the solvent. According to the stability of the droplets, the measurements were taken over a period of 120 s.

### 2.3. Synthesis of Dianhydride Monomer 3FPODA

**Scheme 1:**
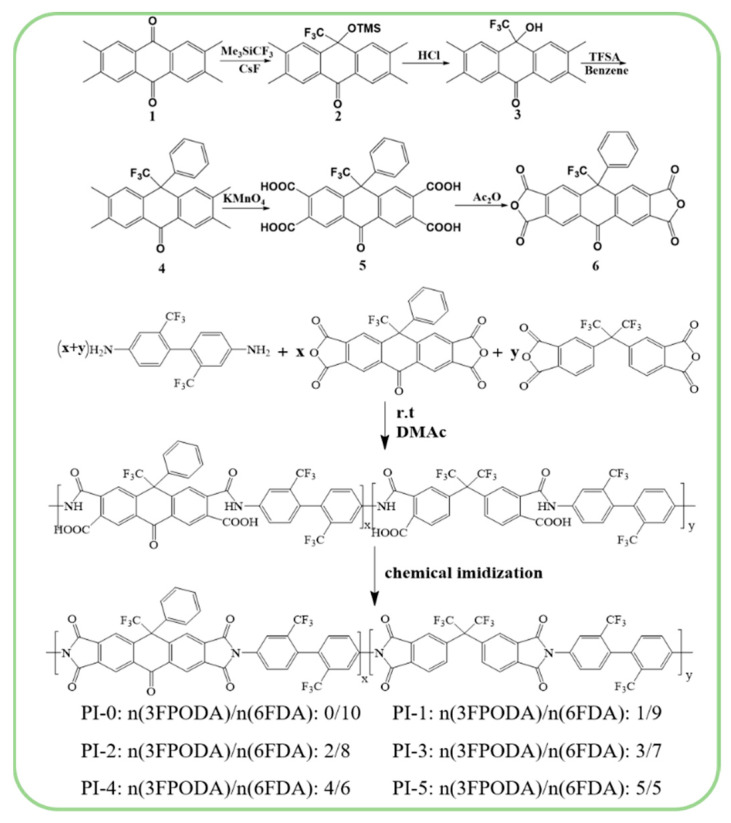
The route of the preparation of 3FPODA, and the synthesis of copolymerized polyimid.

The dianhydride, 10-oxo-9-phenyl-9-(trifluoromethyl)-9,10-dihydroanthracene-2,3,6,7-tetraacid dianhydride (3FPODA), was prepared though five-step route as shown in Scheme 1.

(1) 2,3,6,7-tetramethylanthracene-9,10-dione (10.00 g; 37.83 mmol; 1.00 equivalent) was deposited into a 1000 mL three-necked flask under the protection of nitrogen; then, the CsF (57.5 mg; 0.043 mmol; 0.01 equivalent) and 500 mL of anhydrous tetrahydrofuran were added into the flask. The reaction solution was cooled to −10 °C, and after stirring for 30 min, trimethyl(trifluoromethyl)silane (6 mL, 39.79 mmol, and 1.00 equivalent) was added. After continual stirring for 30 min, the reaction mixture was slowly heated to 10 °C. Then, the reaction was continued for 3 h. Afterwards, the crude product was obtained through filtering the reaction mixture and washing the filter cake with dichloromethane (50 mL/3 times). The pure product was acquired through separating and purifying the crude product by silica gel (petroleum ether/ethyl acetate: 10/1) column chromatography. 2,3,6,7-tetramethyl-10-(trifluoromethyl)-10-(Trimethylsilyl) oxyanthracene-9(10H)-one (6.23 g, 15.33 mmol, 40.5%). ^1^H NMR (600 MHz, CDCl_3_) δ 7.981 (s, 2H), 7.627(s, 2H), 2.34(d, 12H), −0.185 (s, 9H); ^13^C NMR (600 MHz, CDCl_3_, δ) 182.77, 142.49, 138.49, 136.75, 129.93, 129.39, 128.20, 125.25, 123.17, 20.43, 19.27, 1.51; ^19^F NMR (600 MHz, CDCl_3_) δ −79.624 (s, 3F).

(2) 2,3,6,7-tetramethyl-10-(trifluoromethyl)-10-(Trimethylsilyl) oxyanthracene-9(10H)-one (10 g, 24.6 mmol, and 1.00 equivalent) was deposited in a 250 mL three-necked flask; then, 50 mL of THF was added to dissolve the solid through heating it to reflux temperature. After stirring for 10 min, concentrated hydrochloric acid was added slowly, with a white precipitate forming at this time. Then, we continued to add concentrated hydrochloric acid slowly until the white precipitate no longer increased, and the reaction continued to reflux for 5 h. The crude product was obtained by filtering the reaction mixture after the reaction. Then, the pure product was obtained through washing the filter cake several times with n-hexane water. 10-Hydroxy-2,3,6,7-tetramethyl-10-(trifluoromethyl) anthracene-9(10H)-one (7.53 g, 22.5 mmol, 91.5%) was obtained as a white solid. ^1^H NMR (600 MHz, DMSO) δ 7.878 (s, 2H), 7.796 (s, 2H), 7.572 (s, 1H), 2.364–2.319 (d, 12H); ^13^C NMR (600 MHz, DMSO, δ) 182.81, 143.14, 139.04, 137.31, 130.10, 129.33, 127.54, 125.69, 71.70, 20.48, 19.42; ^19^F NMR (600 MHz, DMSO) δ −78.43 (s, 3F).

(3) TFSA (10 mL) was added to an ice-cooled solution of 10-Hydroxy-2,3,6,7-tetramethyl-10-(trifluoromethyl) anthracene-9(10H)-one (5 g, 15 mmol, and 1.00 equivalent) in dry benzene (15 mL). Stirring was continued at 0–5 °C for 2 h and the mixture solution was warmed room temperature; then, the stirring was continued for 24 h, followed by the usual aqueous workup. The crude product was obtained by evaporating the solvent. The crude product was recrystallized using dichloromethane as a good solvent and ethanol as a poor solvent to obtain colorless crystals: 2,3,6,7-tetramethyl-10-phenyl-10-(trifluoromethyl) anthracene- 9(10H)- one (5.52 g, 14 mmol, 93.3%.). ^1^H NMR (600 MHz, CD_2_Cl_2_) δ 8.1 (m, 2H), 7.353–7.303 (m, 5H), 6.843 (m, 2H), 2.357–2.202 (m, 12H); ^13^C NMR (600 MHz, CD_2_Cl_2_, δ) 180.14, 139.75, 138.33, 135.39, 135.17, 129.86, 127.78, 126.33, 125.61, 124.66, 123.91, 122.02, 53.98, 17.23, 15.80; ^19^F NMR (600 MHz, CD_2_Cl_2_) δ −69.53 (d, 3F).

(4) 2,3,6,7-tetramethyl-10-phenyl-10-(trifluoromethyl) anthracene- 9(10H)- one (5 g, 12.7 mmol, 1.00 equivalent) was deposited in a 500 mL three-necked flask, 160 mL of pyridine and 40 mL of water were added, and then the solid of the mixture was dissolved by heating it to reflux temperature. Potassium permanganate (20 g, 127 mmol, and 10.000 equivalent) was then added into the mixture portion wise. The reaction was continued for 16 h; then, the crude product was obtained by filtering the reaction mixture through hot water, concentrating the filtrate to 50 mL, and adding an appropriate amount of concentrated hydrochloric acid to the concentrated filtrate. Then, the pure product, 10-oxo-9-phenyl-9-(trifluoromethyl)-9,10-dihydroanthracene-2,3,6,7-tetracarboxylic acid, was obtained through recrystallizing the crude product with acetic acid. The yield of this step was 80%. ^1^H NMR (600 MHz, DMSO) δ 14.193–13.200 (s, 4H), 8.566 (s, 2H), 7.573–7.411 (m, 5H), 7.319 (s, 2H); ^13^C NMR (600 MHz, DMSO, δ) 180.85, 167.79, 167.56, 141.79, 138.76, 137.52, 134.01, 133.34, 132.03, 129.88, 129.36, 127.71, 126.16, 124.27, 57.06. ^19^F NMR (600 MHz, DMSO) δ −65.79 (s, 3F).

(5) 10-oxo-9-phenyl-9-(trifluoromethyl)-9,10-dihydroanthracene-2,3,6,7-tetracarboxylic acid (5 g, 9.73 mmol, and 1.00 equivalent) was deposited into a 100 mL three-necked flask, 30 mL of acetic anhydride was added, and the solid of the mixture was dissolved by heating it to reflux temperature. Then, the reaction was continued for 3 h. The crude product was obtained through concentrating the reaction solution. The pure product (3FPODA) was obtained by recrystallizing the crude product with acetic anhydride. The yield of this step was 80%. ^1^HNMR (600 MHz, DMSO) δ 8.797 (s, 2H), 7.561–7.542 (m, 5H), 7.505 (s, 2H). ^13^C NMR (600 MHz, DMSO, δ) 180.64, 162.37, 162.13, 145.68, 138.34, 137.35, 135.55, 132.74, 130.27, 129.86, 129.54, 128.20, 124.27, 58.69. ^19^F NMR (600 MHz, DMSO) δ −65.37 (s, 3F).

### 2.4. Preparation of PI Films

The copolymerization of CPI films with different dianhydride ratios derived from 3FPODA, 6FDA, and TFDB was prepared through traditional two-step method. The structures of the copolymerized polyimides are shown in Scheme 1. With PI-1 as an example, the specific experimental steps were as follows: TFDB (1.000 g and 3.123 mmol) was deposited in a 50 mL three-necked flask; then, 18 g dried DMAc was added to dissolve the TFDB under the protection of nitrogen conditions. Then, 3FPODA (0.149 g and 0.312 mmol) and 6FDA (1.248 g and 2.811 mmol) were added to the reaction in several portions. Then, stirring of the reaction was continued for 24 h at room temperature. After polycondensation reaction, the Poly (amic acid) (PAA) was obtained. Then, an appropriate amount of acetic anhydride/triethylamine mixture was added to the PAA solution and reacted at 60 °C for 24 h. Consequently, the incompletely cyclic PI solution was obtained. The PI film was obtained by casting the PI solution on a clean glass plate. Then, the coated PI was heated at 80 °C for 1 h to release the solvent, and then sequentially heated at 100 °C for 30 min, 150 °C for 30 min, 200 °C for 30 min, 250 °C for 30 min, and 300 °C for 30 min. IR (cm^−1^, film): 1787 (C=O asym. stretching), 1726 (C=O sym. stretching), and 1364 (C–N stretching). The FT-IR spectrum of these PIs are shown below.

## 3. Results and Conclusion

### 3.1. Synthesis and Characterization of Monomer

**Figure 1:**
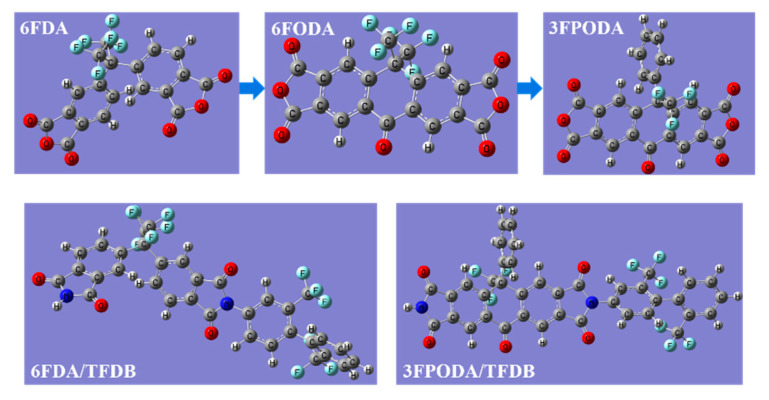
The molecular simulation of monomer 3FPODA, 6FDA, and structural units 3FPODA/TFDB and 6FDA/TFDB.

A new rigid semi-alicyclic dianhydride 3FPODA was prepared through five-step synthesis as shown in Scheme 1, including trifluoromethylation, deprotection, phenyl substitution, oxidation, and a dehydration reaction. The geometrical optimization of the monomers 6FDA and 3FPODA was performed using the density functional theory (DFT) at the B3LYP level with the 6-31G (d) basis set and D3 dispersion correction (OPT B3LYP/6-31G(d) EM=GD3BJ) in the Gaussian 16 program. The molecular simulation of the monomers 3FPODA and 6FDA are shown in Figure 1. The monomer 6FODA was obtained by introducing a carbonyl group to the 6FDA, as shown in Figure 1. Compared with 6FDA, the backbone of the 6FODA molecule is approximately in plane due to the introduction of carbonyl groups. Two -CF_3_ side group could increase the molecular chain’s spacing. Moreover, replacing one of the -CF3 of 6FODA with a benzene ring could result in 3FPODA. Compared with -CF3, the benzene ring has a larger volume and stronger rigidity, thereby further increasing the molecular chain spacing. The structure of 3FPODA was confirmed by FT-IR, ^1^H NMR, ^13^C NMR, and ^19^F NMR spectroscopy (Figures S1–S16). In the ^1^H NMR spectrum, there were three peaks at 8.797, 7.561–7.542, and 7.505 ppm. The peak of the benzene ring side group protons was found at 7.561–7.542 ppm and the peaks at 8.797 and 7.505 ppm were attributed to the protons of the benzene ring on the backbone. In the ^13^C NMR spectrum, all the signals of the carbons were observed at 58–181 ppm. In addition, in the ^19^F NMR spectrum, the peak of a fluorine atom was shown at −65.37 ppm. In the NMR tests, all the observed peaks were consistent with the structure of 3FPODA.

### 3.2. Preparation and Characterization of CPIs

**Figure 2:**
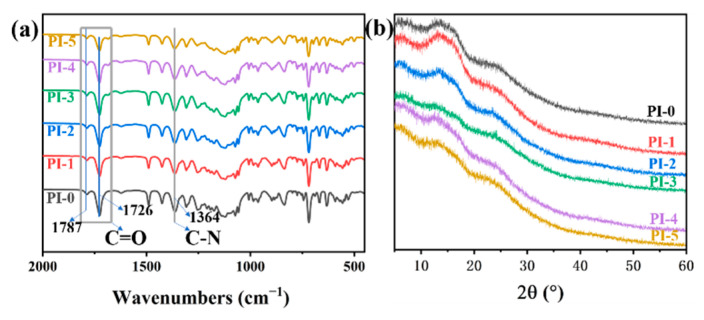
(**a**) The FT-IR spectrum of CPIs; (**b**) The WARD spectrum of CPIs.

A series of CPIs with 3FPODA ratios of 0, 10%, 20%, 30%, 40%, and 50% were coded as PI-0, PI-1, PI-2, PI-3, PI-4, and PI-5, respectively, which were prepared by different ratios of dianhydride from 3FPODA and 6FDA, as well as TFDB as the diamine by a traditional two-step method (Scheme 1). The molecular simulation of the repeating units 3FPODA/TFDB and 6FDA/TFDB are shown in Figure 1. It was found that the repeating unit 6FDA/TFDB has a more twisted structure, while the repeating unit 3FPODA/TFDB has a higher degree of linearity and rigidity of the main chains. The structures of the PIs were confirmed by FT-IR spectroscopy as shown in Figure 2a. The asymmetric and symmetric stretch peaks of C=O of all the CPIs can be clearly observed around 1787 cm^−1^ and 1726 cm^−1^, and C–N stretching can be found around 1364 cm^−1^. The characteristic peaks of amino and carboxyl groups were basically invisible, which indicated that the imidization was finished.

### 3.3. Solubility and WAXD

All the levels of solubility of the CPIs were tested in different organic solvents, as shown in Table S1. At room temperature, these CPIs displayed an outstanding solubility in polar solvents such as NMP and DCM. As listed in Table S1, the solubility levels of the PI-1 to PI-5 were as good as PI-0. Some researchers have revealed that it is possible to increase the free volume of the molecular chain and increase the distance between the molecular chains by introducing solubilizing groups (such as a –CF_3_ group, carbonyl group, and benzene ring side group) in the main chain. These features of the structures facilitate the easier entrance of small solvent molecules into the molecules and improve their solubility [36,37,38,39]. Therefore, the copolymerized CPIs also have an excellent solubility.

The wide-angle X-ray diffraction (WAXD) test was carried out to further study the change in the average spacing distance of the PIs; the results are shown in Figure 2b. These PI films were amorphous, similar to typical PIs. Based on Bragg’s equation, 2*d*sinθ = *n*λ (*n* = 1, λ = 1.54 Å) [35], the average spacing distance (d-spacing) could be calculated according to the WAXD peaks. The results are listed in Table S1. The PI-0 to PI-5 possessed d-spacing values in the range of 6.41–6.65 Å. It can be intuitively found that with the increased amount of 3FPODA, the d-spacing of the PIs increased, which agrees with the theory mentioned above.

### 3.4. Optical Properties-1

**Figure 3:**
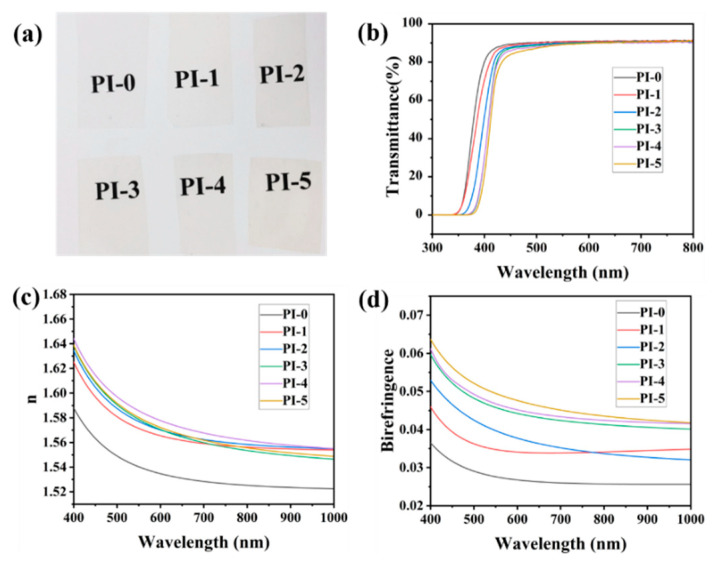
(**a**) The picture of the CPIs; (**b**) The UV–Visible spectrum of CPIs; (**c**) The average refractive index (400–1000 nm) of CPIs; (**d**) The refractive index (400–1000 nm) of CPIs.

A high transparency and low YI are the crucial properties of CPI for the fabrication of transparent FPCBs. A picture of these CPIs is shown in Figure 3a. It is obvious that all the PIs are basically colorless. The optical properties of the PIs were measured by UV–Vis spectroscopy. The UV–Visible spectrum of the PIs is shown in Figure 3b. Their crucial optical properties, including their transmittance at 400 nm (T_400_) and 550 nm (T_550_), cut-off wavelengths (λ_0_), yellow index (YI), and b*, are listed in Table 1. As shown in Table 1, PI-1 to PI-5 exhibited a T_400_ from 30% to 71%, a T_550_ from 89% to 91%, λ_0_ from 343 nm to 372 nm, and a YI from 2.83 to 8.63. However, the contrast PI-0 exhibited a T_400_ of 80%, T_550_ of 91%, and λ_0_ of 345 nm and YI 2.04. Compared with PI-0, the cut-off wavelength of PI-1 to PI-5 was red-shifted, the transmittance at 400 nm decreased significantly, and the YI value also increased. In addition, with the increased of 3FPODA content, the change was more obvious. This may be caused by the carbonyl group of the 3FPODA. In the molecular simulation of 3FPODA, as shown in Figure 1, the carbon atom of the carbonyl group was sp^2^-hybridized, forming a conjugate with the benzene ring on both sides. On the one hand, the existence of this conjugated structure could increase the CTC effect, and the absorption peak of the CTC effect was located in the 360–500 nm band [40,41]. As a result, the YI value of the PIs was increased and the T_400_ decreased. On the other hand, the π electrons of the conjugated structure underwent electronic transitions, and their energy absorption peaks were located in the 300–380 nm range [42,43], which could red-shift the cut-off wavelength of the CPIs. Therefore, it is crucial to control the amount of 3FPODA to prepare CPIs suitable for transparent FPCBs. It is notable that PI-1 to PI-4 possessed better optical properties and the suitable proportion of 3FPODA should be below 40%.

Furthermore, the refractive indices and the out-of-plane birefringence of the CPIs were tested and are shown in Figure 3c,d. The results were summarized in Table 1. PI-1 to PI-5 displayed refractive indices and birefringence at 550 nm in the range of 1.571–1.580 and 0.0347–0.0496, respectively. However, the contrast PI-0 possessed refractive indices and birefringence at 550 nm of 1.540 and 0.0276, respectively. The refractive indices and birefringence of PI-1 to PI-5 were higher than the contrast PI-0, and increased with the increasing proportion of 3FPODA. This was due to the structure of 3FPODA, which contains linear semi-alicyclic structures and a certain conjugation between the carbonyl and benzene ring on both. Its features increased the regularity of the molecular chain of the PIs, provided them with a higher orientation, and increased their birefringence.

### 3.5. Thermal Properties

**Figure 4:**
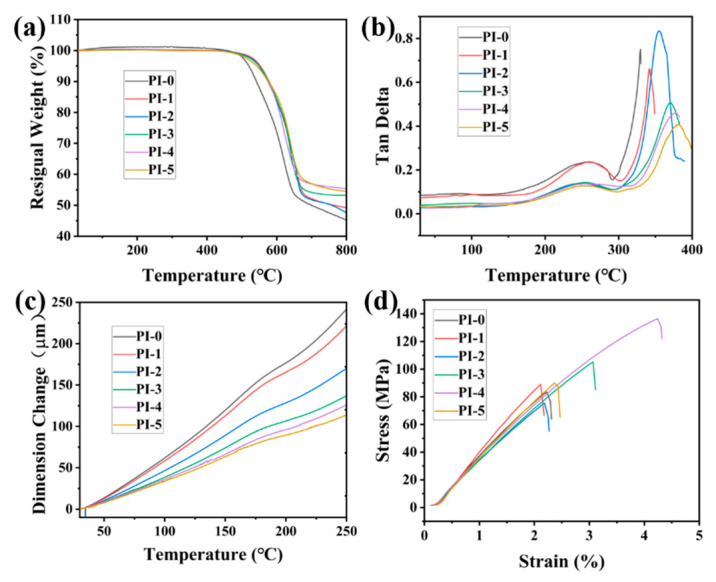
(**a**) The TGA curves of CPIs; (**b**) The DMA curves of CPIs; (**c**) The TMA curves of CPIs; (**d**) The stress–strain curves of CPIs.

The thermal properties, including the T_g_ values and CTE values, were the critical indexes governing the ability of the CPIs to satisfy the process of creating a transparent FPCB. The thermal properties of these CPIs were evaluated by thermogravimetric analysis (TGA), dynamic mechanical analysis (DMA), and thermal mechanical analysis (TMA). The results are shown in Figure 4, and the thermal indices (T_g_, T_d1_, T_d5_, and CTE) are listed in Table 2. PI-1 to PI-5 possessed T_g_ values in the range of 342–382 °C, while the T_g_ of the contrast PI-0 was 330 °C. It can be found from the DMA curves in Figure 4b that the T_g_ of all the copolymerized PIs were higher than the reference PI-0, and the T_g_ of the CPIs increased along with the increase in the amount of 3FPODA. This evidenced that the addition of 3FPODA could increase the T_g_ of CPIs. As shown in Figure 1, the repeating unit of 3FPODA/TFDB possessing a semi-alicyclic ring structure in its backbone enabled it to be more rigid than the repeating unit of 6FDA/TFDB. Importantly, the T_g_ of the CPIs depends on the degree of rigidity of the molecular chain and the CTC effect between the inter or intra molecular chains formed between the dianhydride and diamine residues [44]. The conjugation of 3FPODA between the carbonyl group and the benzene ring next to it could enhance the CTC effect’s influence on the inter or intra molecular chains’ ability to increase the T_g_ of the CPIs. Furthermore, the phenyl side group could restrict the movement of molecular segments and increased the rigidity of the polymer. Thus, the addition of 3FPODA in the CPI ensured that its T_g_ was higher than the PI based on pure 6FDA.

The thermal stabilities of these PIs are shown in Table 2 and Figure 4a. The decomposition temperatures (T_d1_) at a 1% weight loss of PI-1 to PI-5 were in the range of 480–499 °C. In addition, the T_d5_ values were between 542–552 °C. Whereas the T_d1_ and T_d5_ values of the contrast PI-0 were 485 °C and 520 °C, respectively. This indicated that the addition of 3FPODA can improve the thermal stabilities of the PIs. As mentioned above, the rigid structure of 3FPODA enhanced the inter-chain interactions, thereby increasing their thermal stabilities.

The results of the TMA test are shown in Table 2 and Figure 4c. The CTE values of PI-1 to PI-5 were between 23–42 ppm/K. Whereas the CTE value of PI-0 was 46 ppm/K. This demonstrates that the addition of 3FPODA reduced the CTE value of the CPIs and enhanced their dimensional stability. As shown in Figure 1, the semi-alicyclic ring structure and carbonyl group of 3FPODA granted the repeating unit of 3FPODA/TFDB a more rigid and higher linearity than 6FDA/TFDB. Notably, the incorporation of carbonyl group on the backbone prevented the molecular rotation from decreasing the CTE values. Furthermore, the phenyl side group could restrict the movement of the molecular segments to further reduce the CTE of the CPIs [45].

### 3.6. Mechanical Properties

The mechanical properties of the PIs were measured by a tensile test shown in Figure 4d and the mechanical indices (T_S_, T_M_, and E_B_) are summarized in Table 2. PI-1 to PI-5 possessed T_S_ values of 76–136 MPa, E_B_ of 2.05–4.13%, and T_M_ of 3.6–4.5 GPa. As a reference, PI-0 possessed a T_S_ of 84 MPa, an E_B_ of 2.94%, and a T_M_ of 3.2 GPa. From the stress–strain curve, it was found that the addition of 3FPODA enhanced the mechanical properties of the CPI films. As mentioned above, the repeating unit of 3FPODA/TFDB possessed a much more rigid, regular, and highly linear structure than 6FDA/TFDB, which effectively enhanced its mechanical properties [46]. Therefore, the tensile modulus results demonstrated that the increasing amount of 3FPODA increased the rigidity of the PI backbone and significantly enhanced its tensile modulus.

### 3.7. Surface Properties

**Figure 5:**
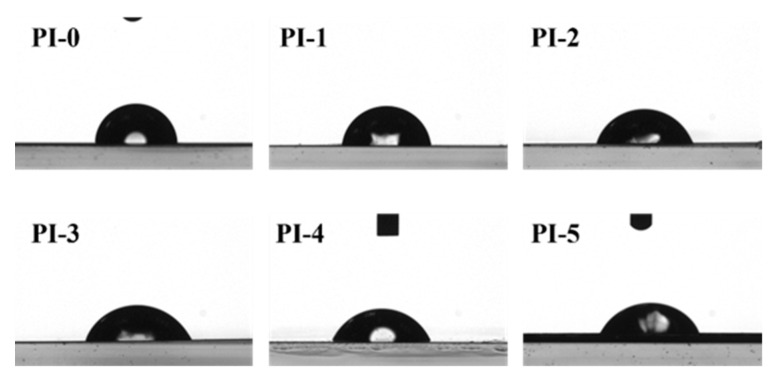
The contact angle of CPIs.

The results of the contact angle test are shown in Figure 5 and Table 2. PI-1 to PI-5 displayed contact angles of 65.4° to 83.3°, and the contact angle of PI-0 was 88.4°. The results demonstrated that the incorporation of 3FPODA enhanced the hydrophilicity of the surface caused by increasing the hydrophilic performance of the polar carbonyl group in the 3FPODA. At present, the commonly used method of preparing the FCCL of the FPCB is to combine PI film with copper foil with an adhesive. The enhanced hydrophilic surface of the CPIs’ surfaces could contribute to the spread and bonding of the adhesive. Moreover, the reactivity of the carbonyl group of 3FPODA could react with the adhesive and further improve the adhesive properties and the bonding strength of the CPI and the adhesive, which could prevent the cracking of the interface.

## 4. Conclusions

In conclusion, a novel, rigid, semi-alicyclic dianhydride, 3FPODA, and a series of modified PIs with different amounts of 3FPODA, were reported. The incorporation of rigid, semi-alicyclic 3FPODA, which possessed -CF_3_ and phenyl side groups and a carbonyl group, contributed to the increased rigidity and linearity of the molecular chain of the CPIs compared to 6FDA. These features endowed the CPIs with better thermal and mechanical properties. According to the copolymerization results, PI-4 possessed the best comprehensive performance with λ_0_ of 371 nm, a T_400_ of 31%, a T_550_ of 90%, a T_g_ of 377 °C, a CTE value of 24 ppm/K, a T_S_ of 136 MPa, an E_B_ of 4.13%, and a T_M_ of 4.4 GPa. These excellent property indexes were crucial for CPIs ability to prepare a transparent FPCB. Moreover, the polar carbonyl group of 3FPODA improved the adhesive properties of the CPIs, which improved the interface inertia of the ordinary PI. Thus, this work provided an effective strategy to modify traditional CPIs towards acquiring outstanding thermal and mechanical properties and good adhesive properties, which satisfies the requirement of a transparent FPCB.

## Figures and Tables

**Table 1:** The optical properties of the CPIs.

PI Film Code	λ_0_^a^ (nm)	T_400_^b^ (%)	T_550_^b^ (%)	b*	YI	n ^c^	∆n_th_^d^	Retardation_th_^e^ /(nm)
PI-0	345	80	91	1.07	2.04	1.540	0.0276	276
PI-1	343	71	91	1.58	2.83	1.571	0.0347	347
PI-2	358	52	90	2.13	3.75	1.577	0.0397	397
PI-3	372	30	90	2.84	4.90	1.579	0.0458	458
PI-4	371	31	90	3.37	5.78	1.586	0.0468	468
PI-5	376	21	89	4.93	8.63	1.580	0.0496	496

^a^ cutoff wavelength of UV–vis; ^b^ transmittance at 400 nm and 550 nm; ^c^ refractive indices at 550 nm; ^d^ out-of-plane birefringence at 550 nm; ^e^ retardation per 10 μm of PI films at 550 nm.

**Table 2:** The thermal properties, mechanical properties, and surface properties of the CPIs.

PI Film Code	Thermal Properties					Mechanical Properties			Surface Properties
	*T*_g_ (°C)	*T*_d1_ (°C)	*T*_d5_ (°C)	Char ^a^ Yield (%)	CTE ^b^ (ppm/K)	*T*_S_ (MPa)	*E*_B_ (%)	*T*_M_ (GPa)	Contact Angle (°)
PI-0	330	485	520	45	46	84	2.94	3.2	88.4
PI-1	342	499	552	49	42	89	2.74	3.6	83.3
PI-2	355	492	549	48	33	76	2.05	4.1	73.8
PI-3	372	489	549	53	27	105	2.93	4.2	70.9
PI-4	377	480	542	55	24	136	4.13	4.4	67
PI-5	382	481	543	55	23	90	2.19	4.5	65.4

^a^ Residual weight retention at 800 °C in N_2_; ^b^ Coefficient of thermal expansion at 50–200 °C; *T*_S_—tensile strength; *E*_B_—elongation at break; *T*_M_—tensile modulus.

## Data Availability

The data presented in this study are available on request from the corresponding author.

## Supplementary Materials

The following supporting information can be downloaded at: https://www.mdpi.com/article/10.3390/polym14194132/s1, Figure S1: The ^1^H NMR spectrum of compound **2**; Figure S2: The ^13^C NMR spectrum of compound **2**; Figure S3: The ^19^F NMR spectrum of compound **2**; Figure S4: The ^1^H NMR spectrum of compound **3**; Figure S5: The ^13^C NMR spectrum of compound **3**; Figure S6: The ^19^F NMR spectrum of compound **3**; Figure S7: The ^1^H NMR spectrum of compound **4**; Figure S8: The ^13^C NMR spectrum of compound **4**; Figure S9: The ^19^F NMR spectrum of compound **4**; Figure S10: The ^1^H NMR spectrum of compound **5**; Figure S11: The ^13^C NMR spectrum of compound **5**; Figure S12: The ^19^F NMR spectrum of compound **5**; Figure S13: The ^1^H NMR spectrum of compound **6** (3FPODA); Figure S14: The ^13^C NMR spectrum of compound **6** (3FPODA); Figure S15: The ^19^F NMR spectrum of compound **6** (3FPODA); Figure S16: (**a**) FT-IR spectra of compound **2**; (**b**) FT-IR spectra of compound **3**; (**c**) FT-IR spectra of compound **4**; (**d**) FT-IR spectra of compound **5**; (**e**) FT-IR spectra of compound **6**; Table S1: The solubility properties of PIs.
