# Supplementary material for: Synthesis of a Novel Rigid Semi-Alicyclic Dianhydride and Its Copolymerized Transparent Polyimide Films’ Properties
Source: Polymers (Basel). 2022 Oct 2;14(19):4132. doi: 10.3390/polym14194132 (PMC9571554; doi:10.3390/polym14194132)
Supplement: Supplementary file 1 [file polymers-14-04132-s001.zip › polymers-1940871-supplementary.pdf]

Synthesis of a Novel Rigid Semi-alicycalic Dianhydride and its Copolymerized Transparent Polyimide Films Properties

Yao Wang, Xiangfu Liu, Jiulin Shen, Jianqiao. Zhao, and Guoli. Tu*

Wuhan National Laboratory for Optoelectronics Huazhong University of Science and Technology 1037 Luoyu Road, Wuhan 430074, China.

Corresponding author E-mail: tgl@hust.edu.cn

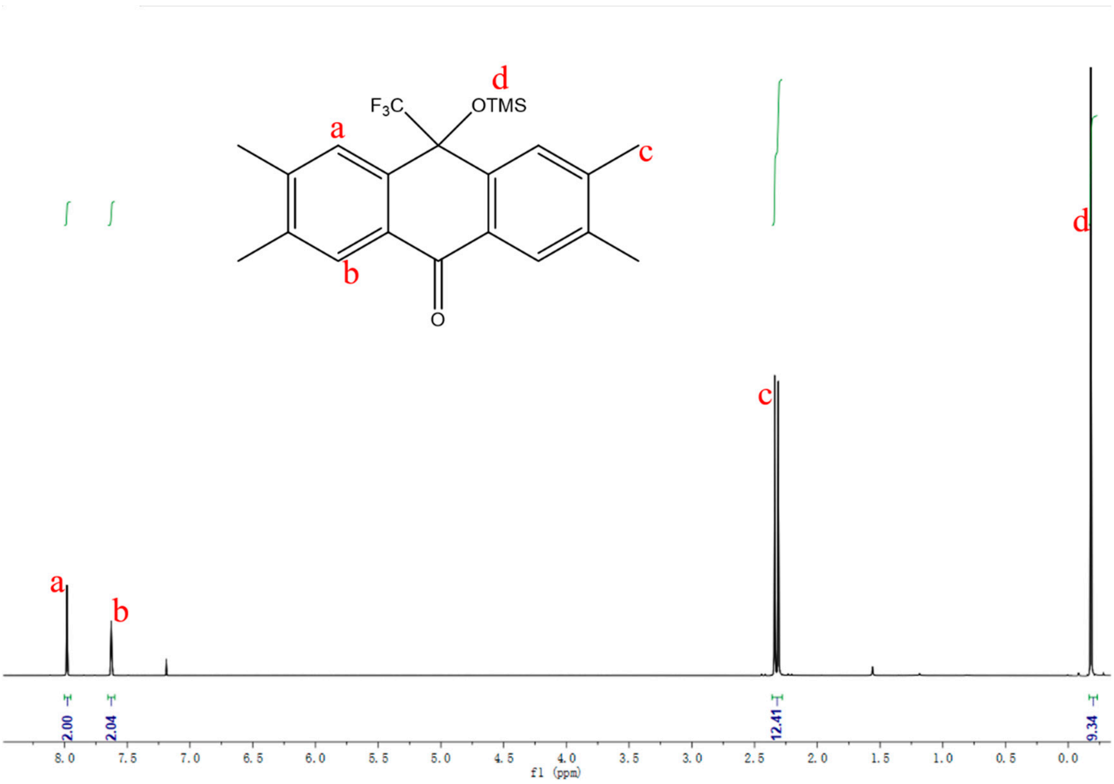

Figure S1. The ^{1}H NMR spectrum of compound 2.

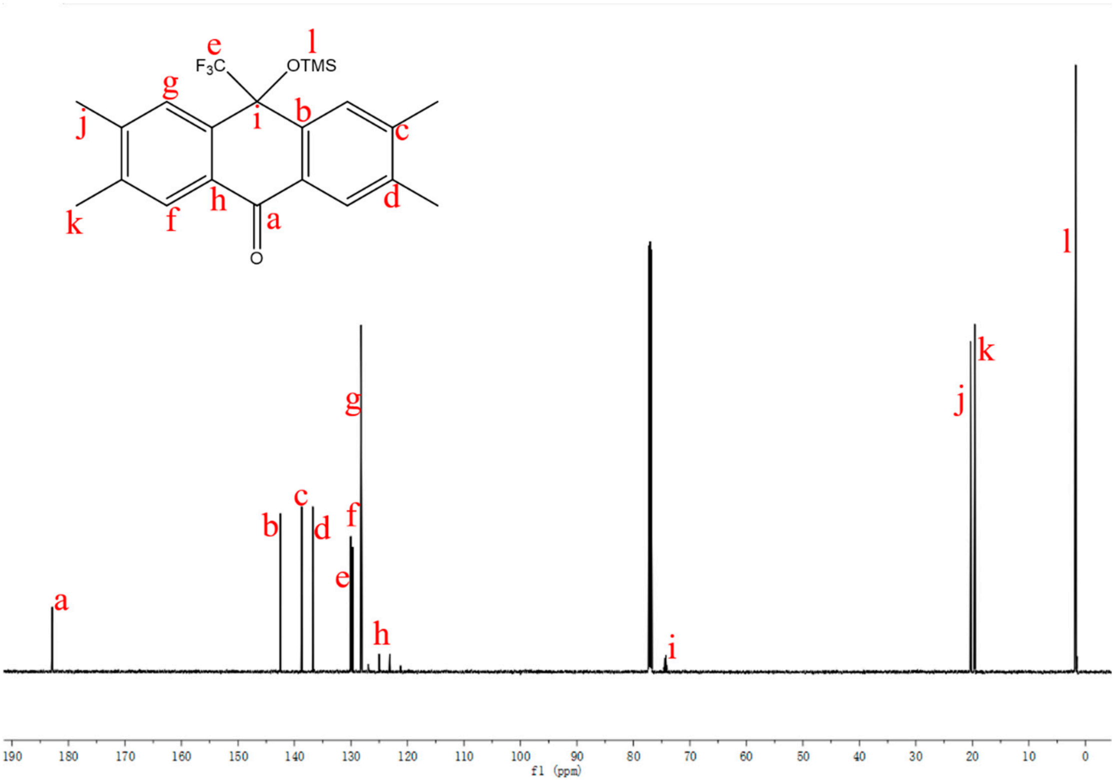

Figure S2. The ^{13}C NMR spectrum of compound 2.

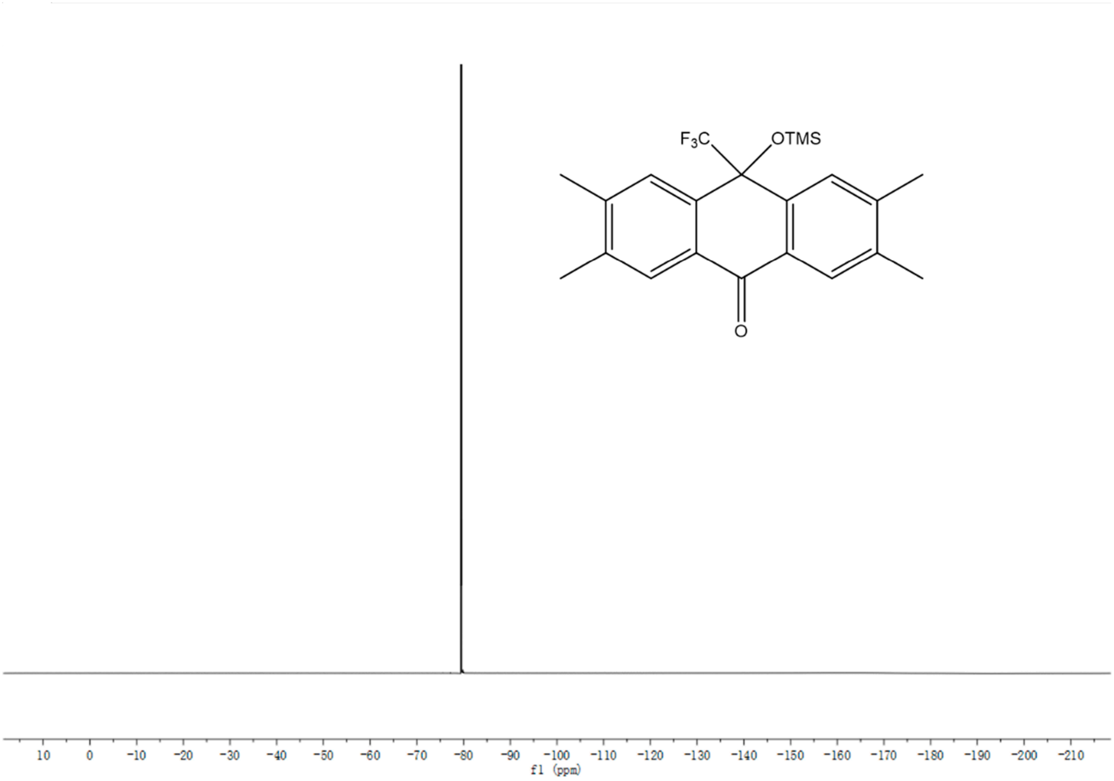

Figure S3. The ^{19}F NMR spectrum of compound 2.

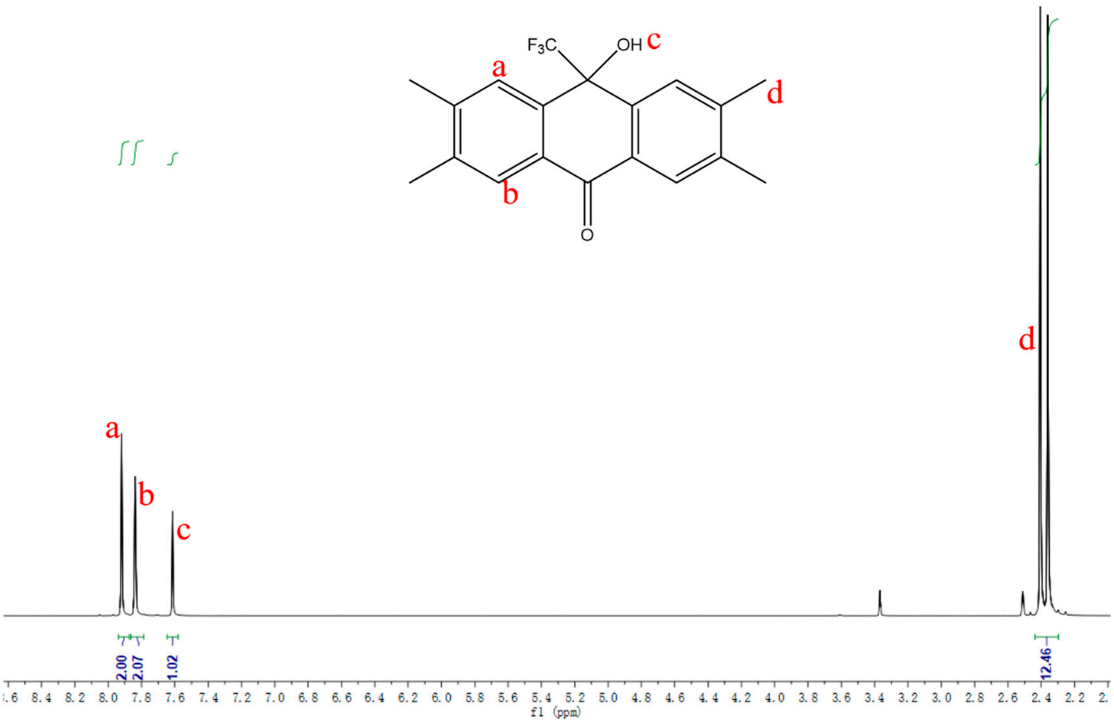

Figure S4. The ^{1}H NMR spectrum of compound 3.

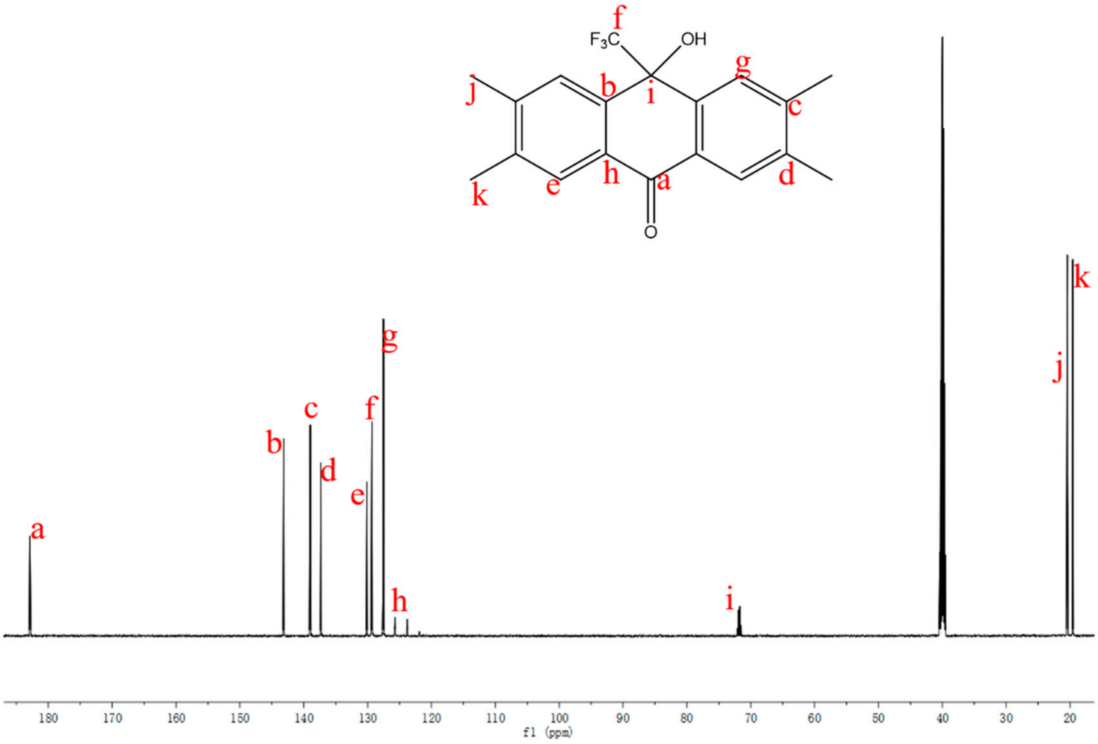

Figure S5. The ^{13}C NMR spectrum of compound 3.

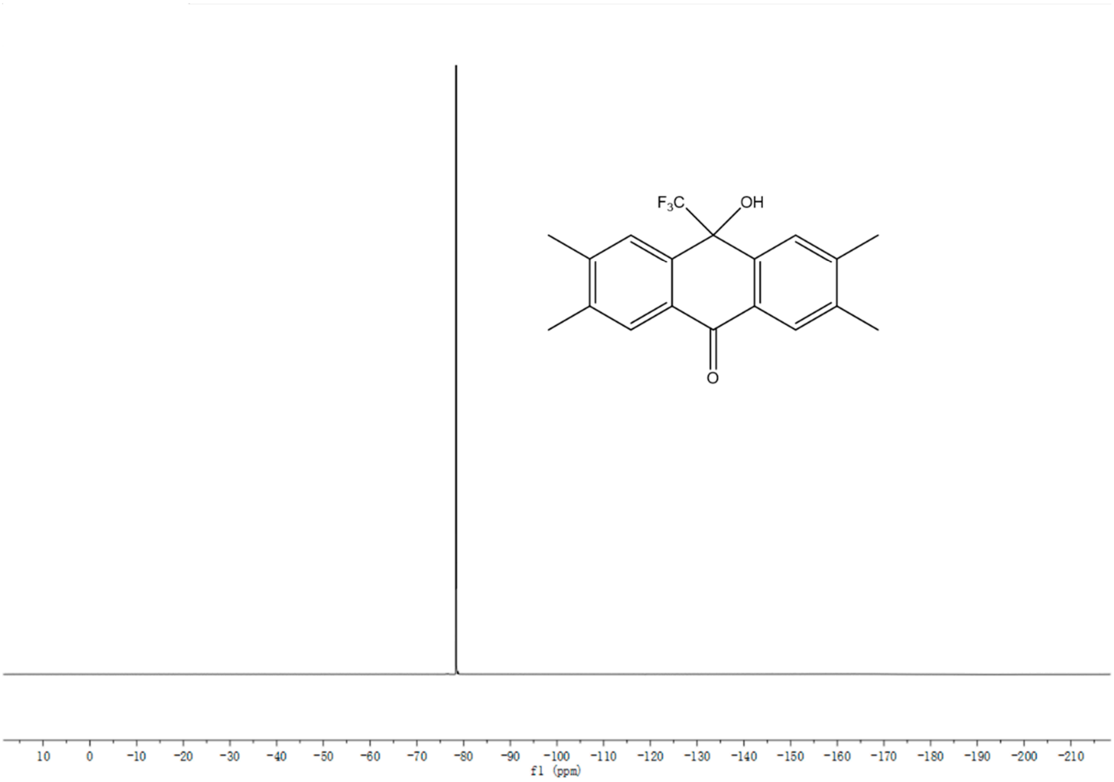

Figure S6. The ^{19}F NMR spectrum of compound 3.

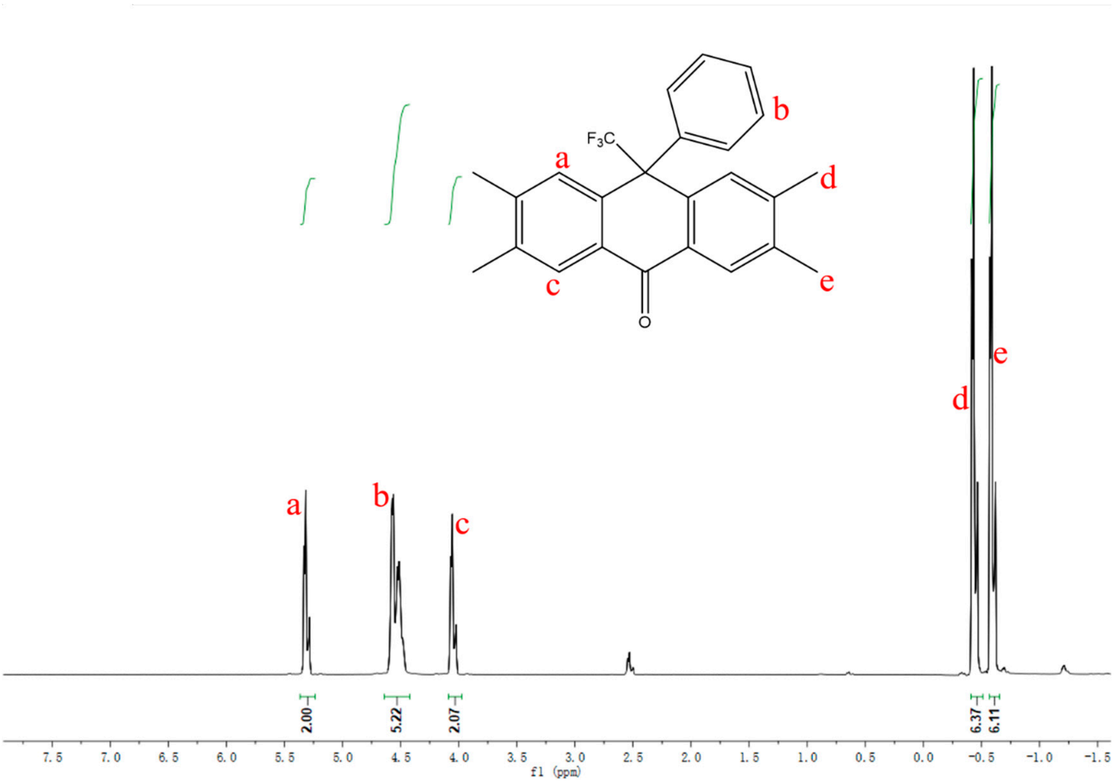

Figure S7. The ^{1}H NMR spectrum of compound 4.

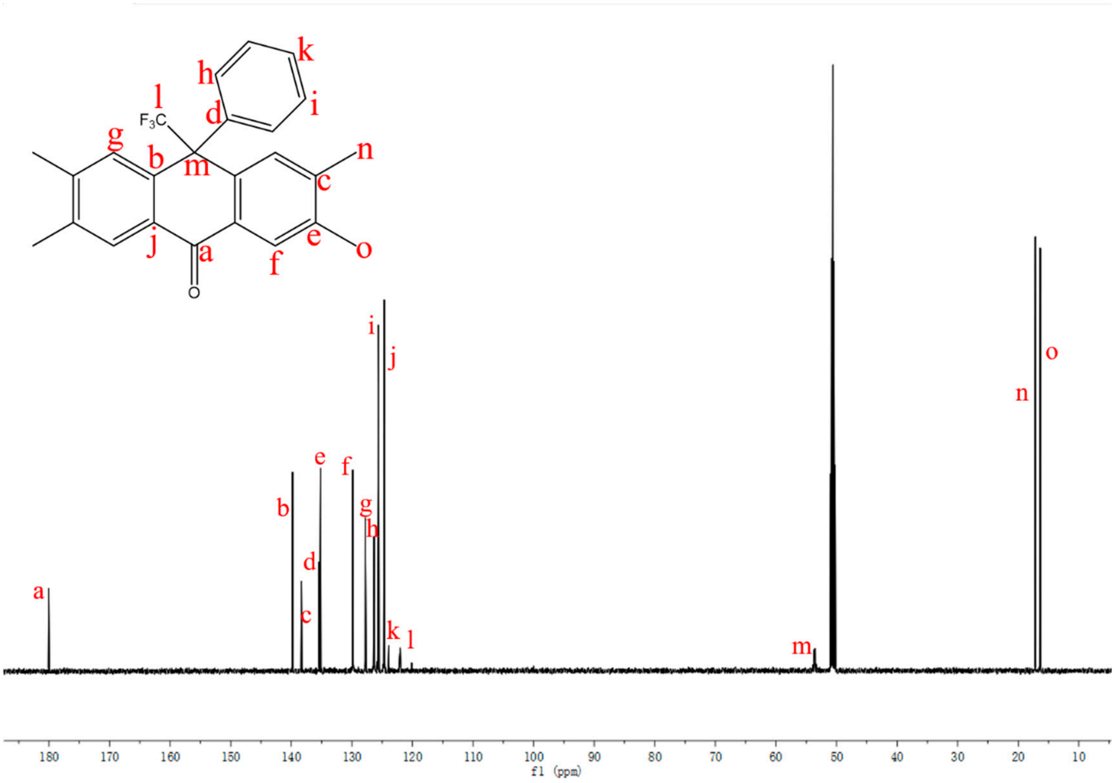

Figure S8. The ^{13}C NMR spectrum of compound 4.

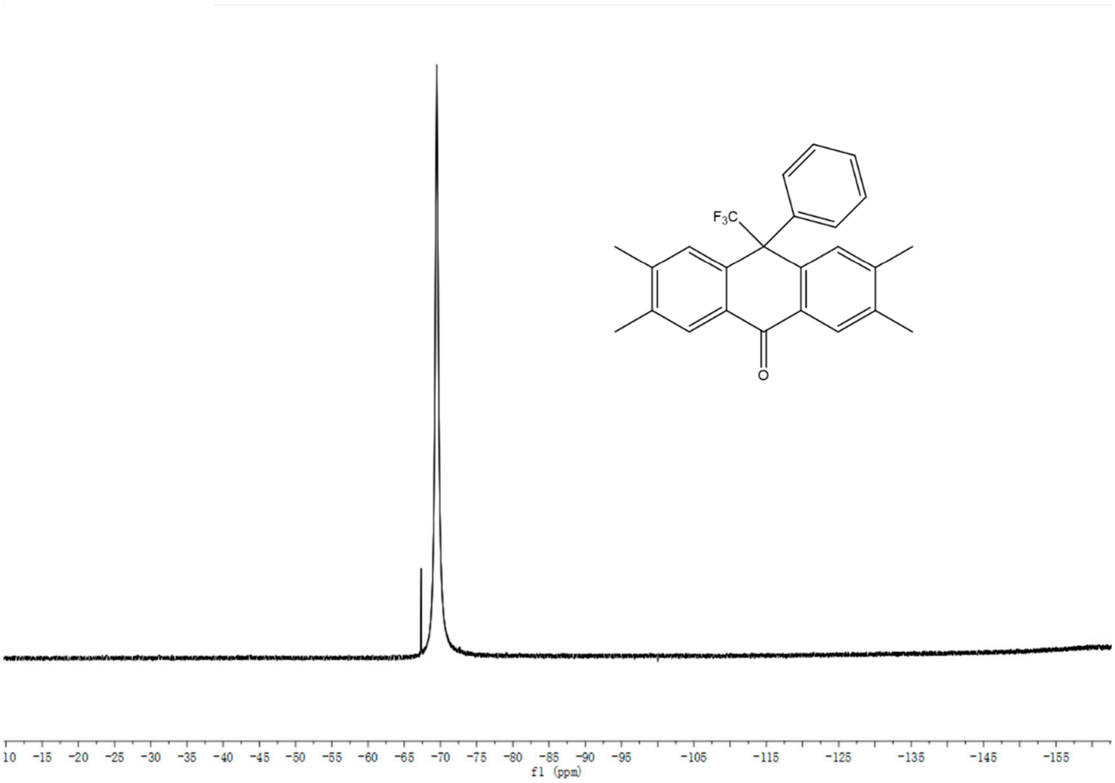

Figure S9. The ^{19}F NMR spectrum of compound 4.

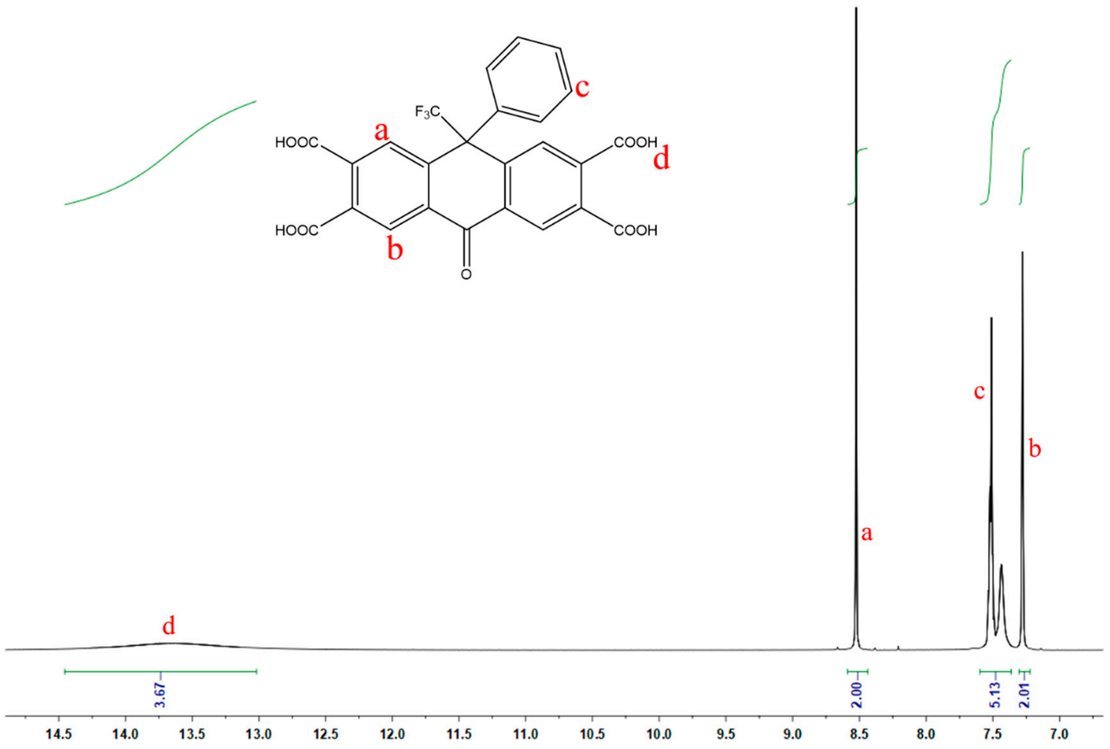

Figure S10. The ^{1}H NMR spectrum of compound 5.

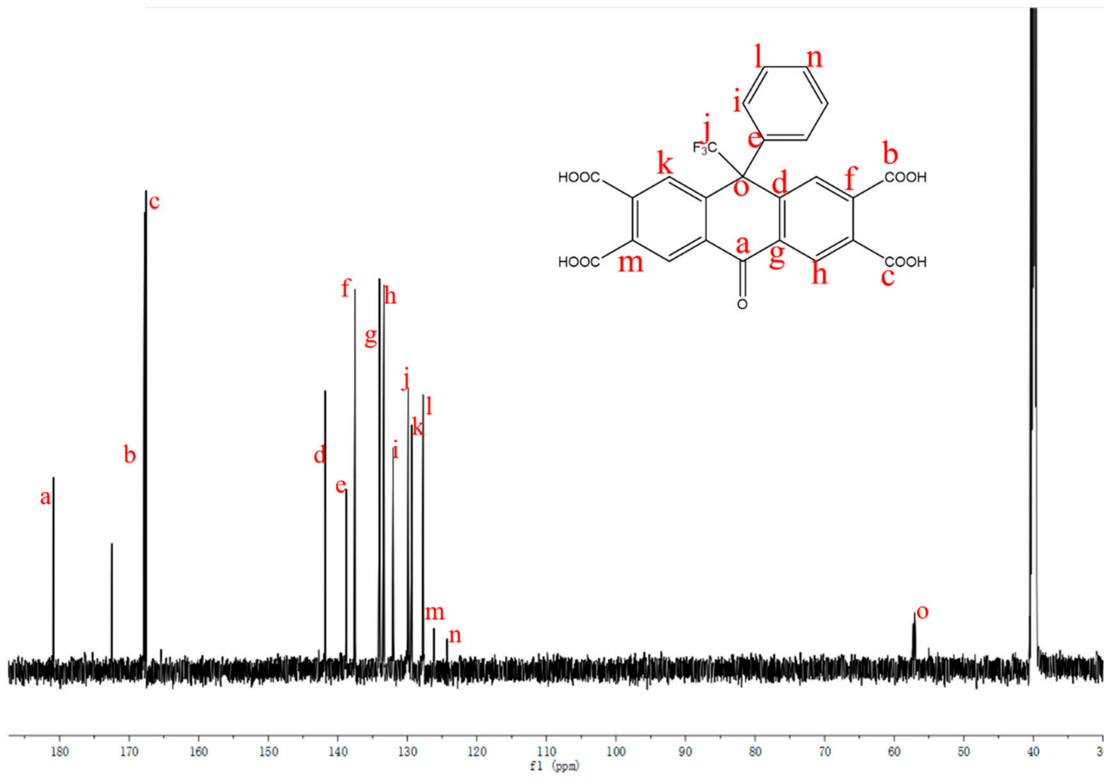

Figure S11. The ^{13}C NMR spectrum of compound 5.

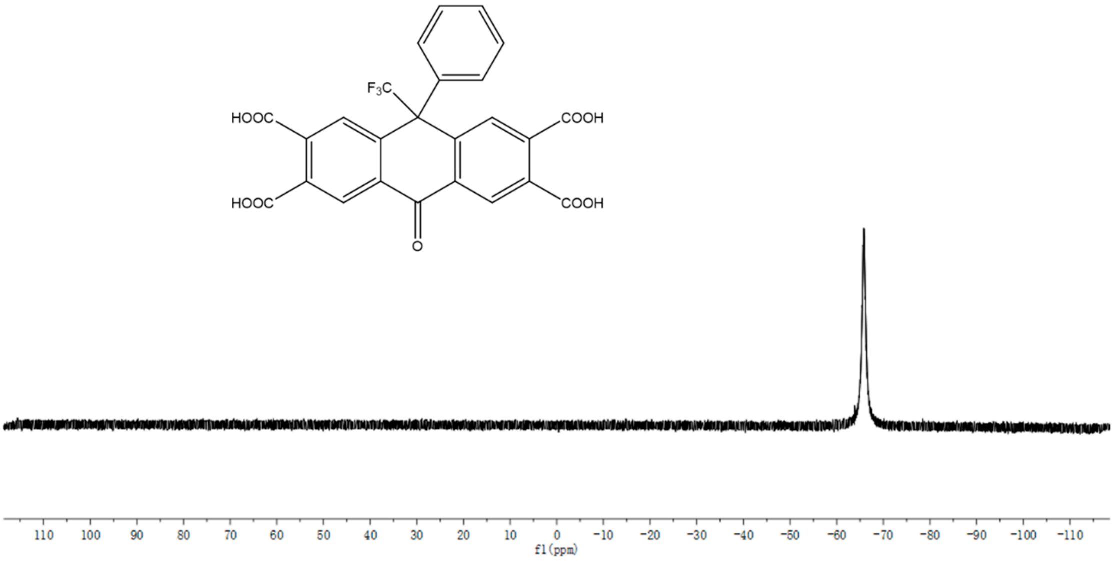

Figure S12. The ^{19}F NMR spectrum of compound 5.

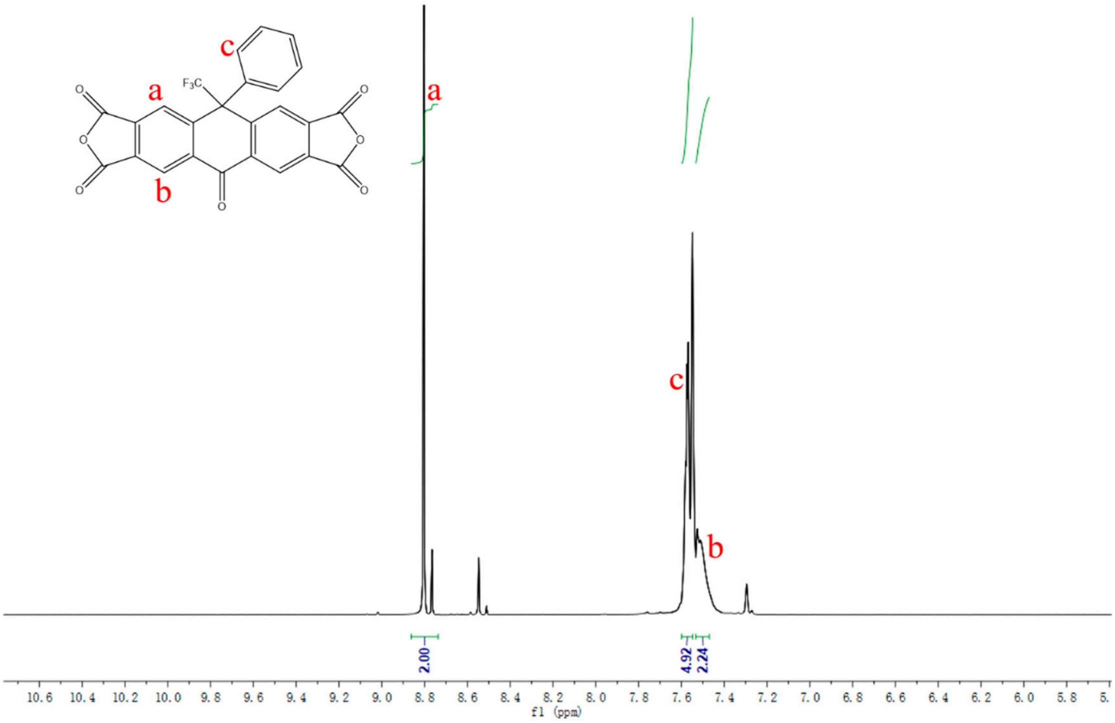

Figure S13. The ^{1}H NMR spectrum of compound 6 (3FPODA).

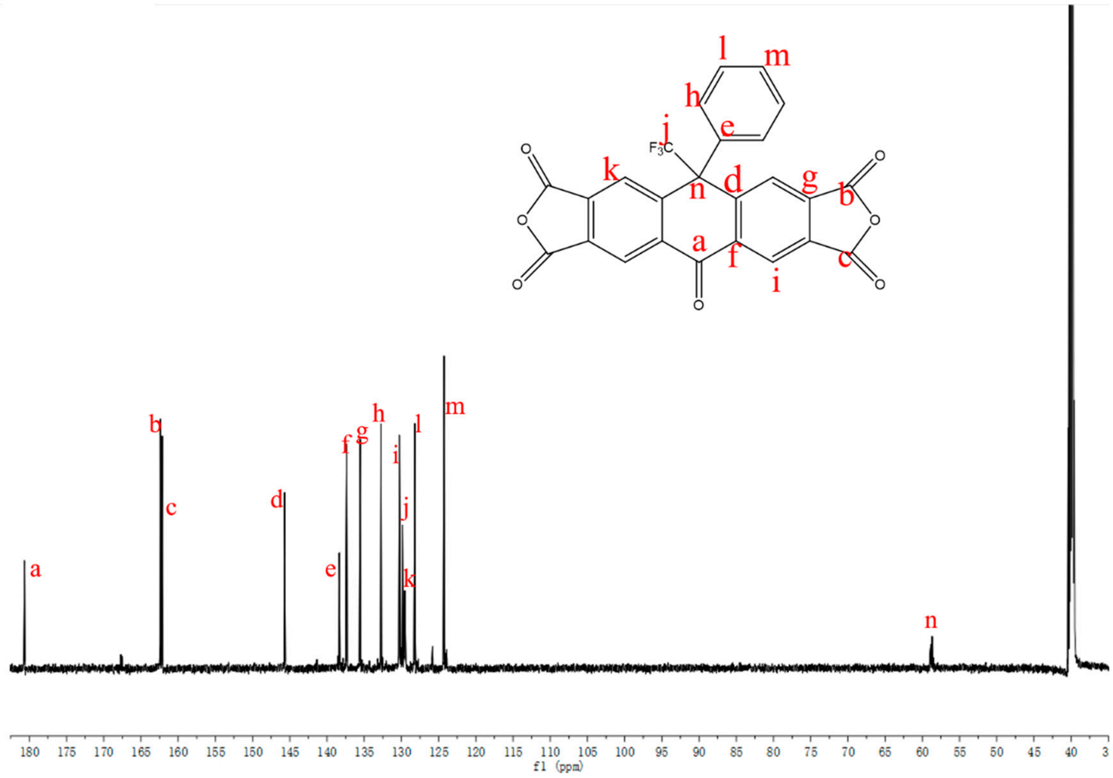

Figure S14. The ^{13}C NMR spectrum of compound 6 (3FPODA).

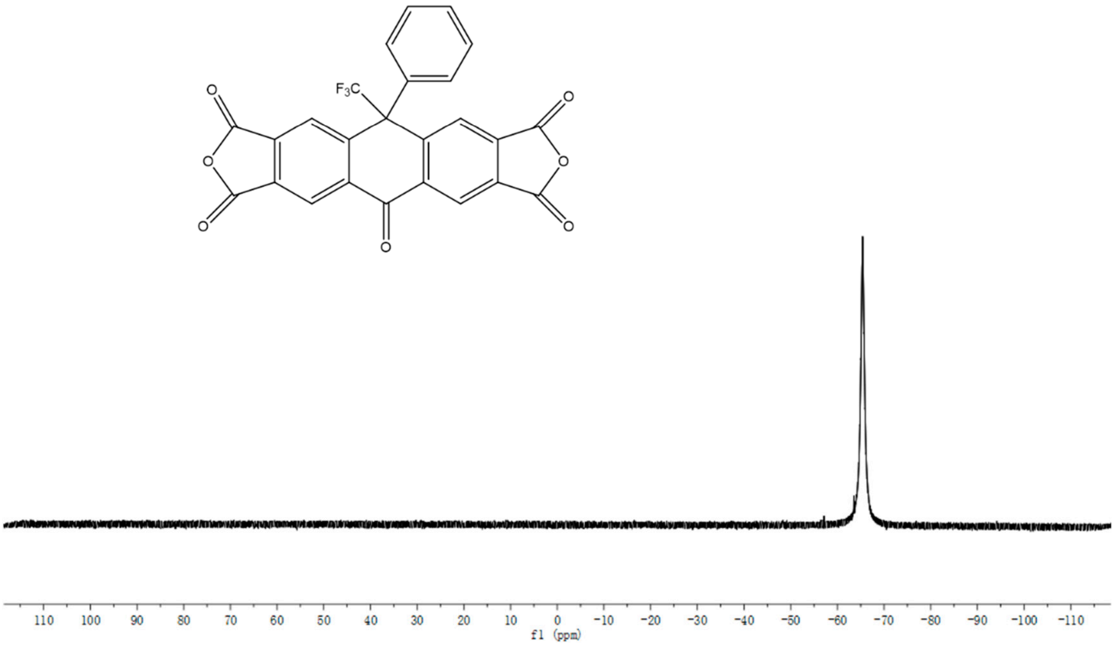

Figure S15. The ^{19}F NMR spectrum of compound 6 (3FPODA).

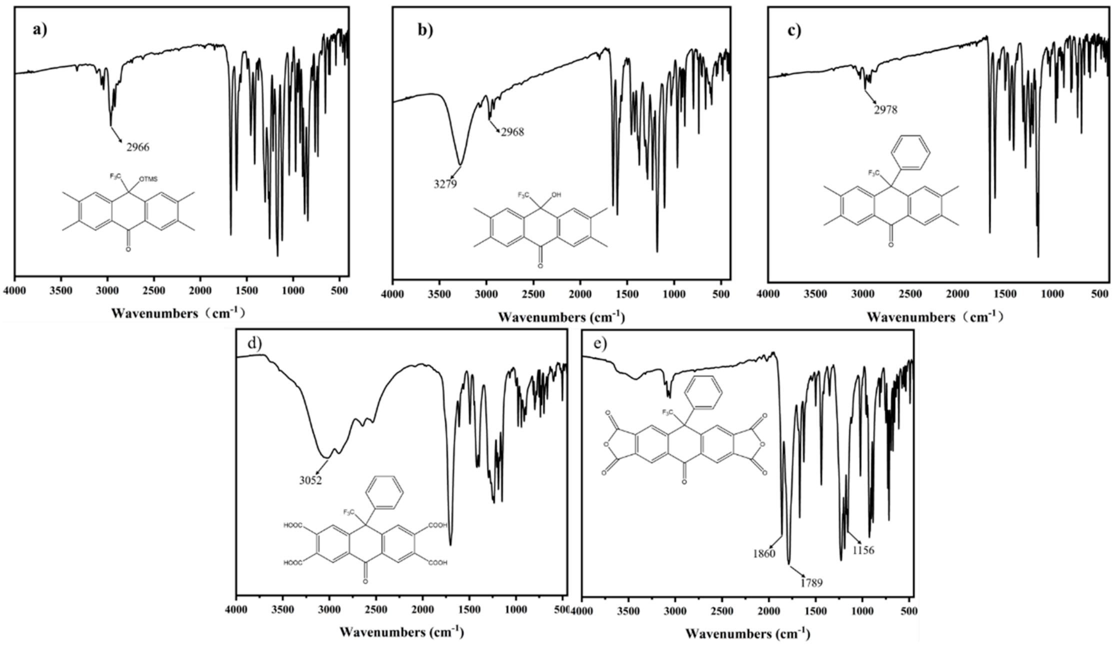

Figure S16. a) FT-IR spectra of compound 2. b) FT-IR spectra of compound 3. c) FT-IR spectra of compound 4. d) FT-IR spectra of compound 5. e) FT-IR spectra of compound 6.

Table S1. The solubility properties of PIs.

PI Film code	Solubility [a]								WAXD	
	THF	EA	Toluene	m-Cresol	DMAc	NMP	DMF	DCM	2θ (◦)	*d*-Spacing (Å)
PI-0	+h	+h	-	+s	+	+	+	+	13.79	6.41
PI-1	+h	+h	-	+s	+	+	+	+	13.71	6.45
PI-2	+h	+h	-	+s	+	+	+	+	13.63	6.49
PI-3	+h	+h	-	+s	+	+	+	+	13.50	6.55
PI-4	+h	+h	-	+s	+	+	+	+	13.25	6.67
PI-5	+h	+h	-	+s	+	+	+	+	13.30	6.65

[a] +, could soluble at r. t.; +h, could soluble with heating; +s; slightly soluble with heating; -, could not soluble
